# Numbers of publications and citations for researchers in fields pertinent to the social services: a comparison of peer-reviewed journal publications across six disciplines

**DOI:** 10.1007/s11192-022-04495-3

**Published:** 2022-08-17

**Authors:** Guy Madison, Knut Sundell

**Affiliations:** 1 Department of Psychology, Umeå University, Umeå, Sweden; 2 Department of Social Work and Criminology, University of Gävle, Gävle, Sweden

**Keywords:** Education, Social science, Citation index, Scientific communication, Research dissemination, Research quality, Sociology, Psychology, Public health, Nursing and caring science, Social work

## Abstract

Timely dissemination of knowledge is essential and fosters development of knowledge both within academe and the wider society, not least for knowledge that serves practises outside of academia. Here, we compare six disciplines which provide health-related knowledge that serve the health and social services. Most previous research compares the size and impact of the body of publications belonging to each discipline, which ignores the distribution of seniority, productivity, and impact amongst researchers. Instead, we consider the whole population of academics in Sweden employed or active within each discipline, including those who have nil publications. The disciplines form three clusters, where researchers in Public Health and Nursing and Caring science claim about 15 articles per author, Psychology about 10, and Education, Sociology and Social Work less than four. Their numbers of citations follow the same pattern, and are substantially correlated with the number of articles. Tenured or full professors had about 50% more publications and citations per publication than had associate professors. The distributions indicate clear modes at 0, 4, and 16 publications for each cluster, and provide the proportions of researchers within each discipline who have no such publications at all. We discuss the implications of these results for policy, practice, and knowledge quality in the social services and the welfare sector.

## Introduction

A central premise of the academic system is effective dissemination of knowledge and ideas, not least through peer review and peer criticism (Righi & Takacs, 2017). The discussion of results and theories plays a central role in maintaining rigour of thought within the academic community and beyond. There are many types of academic publications, like books, book chapters, reports, and conference contributions, but specialised periodical academic journals constitutes the most general and timely system for the exchange of knowledge, whose peer-review process provides exchange and discussion amongst researchers, as exemplified by the journal of Behavioral and Brain Sciences’ target article with open peer commentary.

Publication patterns vary substantially across disciplines. This reflects differing needs and constraints across disciplines, but also to some extent differing historical traditions (cf. Bonaccorsi et al., 2017; Kousha & Thelwall, 2009; Lariviére et al., 2006). In the humanities, scholars are more directly linked to culture and therefore address more often a broader readership, which calls for books rather than scholarly journal articles. Because of their more national or regional orientation they are also more likely to publish in their native language (Nederhof, 2006). One obvious reason for this difference is that disciplines like history, law, and language can be less relevant outside the particular national, cultural, or linguistic domain being studied. Publication rates in peer-reviewed journals, and citations to these publications, are noticeably lower in the social sciences than in the natural sciences, a world-wide trend observed in all countries explored thus far (for reviews see Huang & Chang, 2008; see also Nederhof, 2006). Diana Hicks (1999) argued that social scientists are less pressed by timely publication and by being anticipated by competing researchers, which gives them the possibility to write books, although that takes on average 1.5 years longer than a journal article. Also, social scientists often have an ambition to reach a non-academic audience, such as practitioners and policy makers, for which books might be more appropriate. Accordingly, the social science literature is more fragmented than that of medicine and the natural sciences, argues Hicks, reflecting a lack of reigning international paradigms within the field. This assessment is also supported by more recent research (Huang & Chang, 2008).

However, the social sciences are becoming increasingly internationalised, as reflected in increasing rates of publication in peer-reviewed journals in the Scandinavian countries (Ingwersen, 2000; Kyvik, 2003; Sundell & Åhsberg, 2016). This is concomitant with a global trend towards increasing internationalisation of economies and cultures, and may be partly enforced through EU funding of social science research, as also noted by Hicks (1999). Yet, it has been argued that researchers from different disciplines differ in their choice of research questions, use of methods, and publication patterns, without rational cause (e.g., Olsson & Sundell, 2015), as well as in their productivity as a whole, even though the subject matter is the same and serves the same purpose. The social services seems to be such an area, where highly similar needs and constraints reasonably apply to the extra-academic beneficiaries of the produced knowledge (Sundell, 2010). The purpose of the present study is therefore to describe and quantify such differences for a representative sample of disciplines that serve the health and social services. Here, we include education, nursing and caring science, public health, psychology, social work, and sociology, disciplines that range across medicine and parts of the social sciences.

Previous research along these lines has almost exclusively compared the size and impact of the body of publications belonging to each discipline. Doing so ignores the number of researchers within the compared areas, as well as the distribution of seniority, productivity, and impact amongst researchers. Here, we focus on usefulness and efficacy, and must therefore take the “base rate” of active researchers into account. Otherwise, one apparent problem is that a body of publications will only include individuals who have actually published at least one item within the time frame and the body of publications specified by the inclusion criteria. This would, for example, produce inflated estimates of the productivity of researchers within a certain discipline as a function of the proportion of individuals who have nil publications, as they will not be even considered as potential authors. Thus, a fair comparison of efficacy must involve the whole population of academics employed or active within each discipline. We therefore take the laborious approach of identifying the whole population of active researchers within each discipline, and then measuring the bibliometric output for each one of them, regardless of whether they have actually published anything. Publication databases do not inform about authors’ academic status, and are oblivious as to whether authors constitute graduate students, junior or senior faculty, or some temporary collaborator. Some of this information can be gleaned from their affiliations, but this requires further data collection and is error-prone. Identifying the members of faculty and their seniority provides a fair comparison across disciplines, given the researchers’ competence and available resources, in terms of funding and time for research. To this end, we choose Sweden as a model, because our local knowledge and networks make it possible to identify the population of all researchers who were active in Sweden during a particular time period.

Having thus selected the disciplines to compare and the individual researcher as the unit of analysis, we chose articles in peer-reviewed journal as an index of productivity. This is based on several arguments. As mentioned above, periodical academic journals provide a common and comprehensive system for disseminating knowledge. As such, journal articles are also more available for reliable quantification through cross-disciplinary and searchable databases. Consequently, journal bibliometric data are more stable and reliable than those for books and book chapters, both across fields and across time, as indicated by growth rates (Chi, 2016). They are also the most common publication type across several social sciences disciplines, the publications of which reference on average about 7% edited books and 20% books (Jokic et al., 2019). Across eight European countries, the grand average was estimated to 56.5% journal articles, ranging from 49% in Poland to 77% in Slovakia, with Denmark and Norway close to 60%, two Scandinavian countries neighbouring to Sweden (Kulczycki et al., 2018). In terms of impact, book chapters are lowest, references to specific individual book records have higher impact than the whole books, and individual authored books have the highest citation impact amongst all the document types, a pattern which holds across several fields in chemistry, the biosciences, humanities, and social sciences (Chi, 2016). Thus, authors of one or more books might garner substantial numbers of citations, if they attract attention, and will otherwise not increase their impact at all. Also, books and book chapters constitute an unreliable metric for the present purpose, given that a small proportion of academics author or edit even one book. That being said, ignoring books could induce a bias if there were an inverse relationship, such that those who publish books produce fewer articles. However, there are, in contrast, substantial positive correlations between the number of journal articles and the numbers of monographs and edited books, respectively, across authors and fields (Puuska, 2010, p. 434).

Here, we examine differences in the dissemination of scientific research through peer-reviewed journal articles across disciplines that serve the social services. This focus is motivated in particular by their influence on social policy, practice and culture, and hence by the importance of the quality of knowledge provided (Sundell & Olsson, 2017). These influences are often quite direct, as when informing new legislation and implementing practices. Inasmuch as lively and timely exchange of knowledge and ideas is important for the quality of research, it will also affect the practical usefulness of that research, which has direct and broad effects on individuals’ wellbeing. The effectiveness of the social services hinges upon the continuous development of current knowledge (Sundell & Åhsberg, 2016). Many of the problems they address are subject to sometimes rapid economic, demographic, technical, and other societal changes. Contemporary examples include immigration, criminality, social media, the availability of drugs, and epidemics, like Covid-19. There is also the long-standing global paradigm shift towards the provision of evidence-based social health services, as expressed for example in policy documents from Canada (National Forum on Health, 1997), Sweden (Olsson & Sundell, 2015), and the United States (Honoré et al., 2011), which further accentuates the importance of speed and quality in disseminating research across all relevant disciplines. Because such work may well be published in the native language, we include articles written in both English and Swedish. Again, our purpose is not to compare the relative performance of individuals or institutions within or across disciplines, but to assess actual efficacy. Therefore, it would be counterproductive to employ field normalisation of productivity or impact (e.g., Bornmann et al., 2016; de Rijcke et al., 2016).

In conclusion, the purpose of the present study is to describe, with some precision and in some detail, possible differences in the number of publications and the citations to these publications for researchers within six disciplines that provide health-related knowledge that serve the health and social services. To our knowledge, this comparison has not been made, for any country. Specifically, we compare all researchers who are active at departments that cover these disciplines in Sweden, and control for the possibility that these populations differ in their academic seniority by also considering their academic rank, as expressed in being a professor or an associate professor (docent).

## Methods

### Participants

The target population consists of academics that are expected to perform and publish research. The academic systems and their terminology differs across countries. Swedish academic institutions have two types of positions for PhDs, professor and “lektor”. Professor is the highest academic rank in Sweden, corresponding to tenured or full professor in the USA. Lektor means lecturer and is the most common teaching position, corresponding to assistant or associate professor in the USA. Sweden also has the purely academic title *docent*, corresponding to reader in the UK and associate professor in the USA. The practical consequences of these ranks and positions pertain mainly to the proportion of time available for research in lieu of external research funding. A lecturer typically teaches full time, but may be given up to 20% of for research, which may increase up to 40% for a docent. A professor can usually devote 30–70% to research. As we can therefore not expect lecturers to be active researchers, we limit the study population to professors and associate professors (docent).

### Data collection process

We identified Swedish universities and other higher education institutions with departments with active research in (1) Public Health, (2) Nursing and Caring science, (3) Education, (4) Psychology, (5) Social Work, and (6) Sociology. We defined departments with active research as those who had personnel with a graduate degree in 2009, according to the records of the Swedish National Agency for Higher Education. In total, 99 departments or equivalents were found to be eligible, spread across 27 Swedish universities and institutions of higher education.

We then obtained information on professors and associate professors active at these departments at the beginning of 2010. This resulted in a list of 1046 researchers. Thirty-nine of these were so-called emeritus professors, guest professors, or adjunct professors, meaning they have some non-typical and temporary position at their institution. As they differ considerably amongst themselves, as well as compared to regular professors, all of these were excluded. Some of the remaining 1007 were affiliated with more than one department (e.g., both Public Health and Nursing and Caring science departments), and were assigned to the department which best suited their research profile, as described on their website. In cases where departments comprised researchers active in multiple disciplines, researchers were assigned primarily by their research profile, or, when this was not apparent, by information provided by the head of department. After these assignments, the population consisted of 959 unique individuals, 516 professors and 443 associate professors.

Being compiled in 2012, the data provide a timely description of the then differences between disciplines. We maintain that results based on these data are relevant even ten years later. First, they are representative for the respective academic traditions, which persist to this day. Second, there are no indications of substantial changes in the relation between these disciplines in terms of publication patterns across this period. The productivity of individual researchers seems to be almost constant across time, although the annual rate of scientific publications continuously increases (Fanelli & Lariviére, 2016). Third, these data have the rare properties of following a particular set of individuals over a period of time, and of doing so for an entire population of researchers. To our knowledge, this comparison has not been made, for any country.

Finally, the numbers of publications and citations of those publications were obtained on the Web of Science for each of these researchers for the ten-year period 2000–2009 and independent of the language of the article. The search process proceeded as follows: (1) Search for researcher’s name, (2) check that affiliation matches to confirm it is the right person, and (3) if the researcher had worked overseas or at another institution during the data collection period, the affiliation and abstracts were scrutinized to confirm that it was the same researcher. Double family names (e.g., Andersson-Karlsson, B.) were treated as follows. It was first searched for following steps 1–3, and then all possible name and initial combinations were also searched (e.g., Andersson, B., Karlsson, B., Andersson, K.B., Andersson, B.K., Karlsson, A.B., Karlsson, B.A.). In effect, we controlled for multiple affiliations, spelling of the institution name, and multiple family names and name changes (e.g., due to marriage) (van Raan, 2005). We did not check for misspelling of the author name, but because such errors are randomly spread across disciplines and academic title they are unlikely to affect our results. We did also not distinguish self-citations or different types of articles, such as primary/original research articles, reviews, editorials and conference presentations. However, the latter three publication types were uncommon for all researchers.

### Considerations for using the Web of Science

Our reasons for using the Web of Science (WoS) include that it accurately reflects scientific impact, even for the social sciences (Bornmann et al., 2016). The proportions of these literatures that is found in WoS has increased substantially across the last 20 years, and was in 2011 around 60% for Education, 85% for Psychology, and 53% for Social and Behavioural sciences (interdisciplinary), and 57% for Sociology (van Leeuwen, 2013, Fig. 4). Figures for health related fields were only available for 2010, and were above 80% for Biological sciences, Basic Medical Science, Clinical Medicine, and Biomedical sciences, and 68% for Health Sciences (van Leeuwen, 2013, Fig. 2). The total number of indexed journals in Web of Science varies, with journals constantly being removed and added, but it presently covers more than 12,000 scientific journals (Testa, 2009). Approximately 2000 new journals are assessed annually and 10–12% of these are chosen for inclusion in the database. The criteria include having peer-review, bibliographic information in English (title, abstract, keywords), and international diversity of authorship and citation data (Testa, 2009). In effect, it includes journals with the highest impact factors, that is, the average number of citations to articles published under the past two years. According to the Web of Science, relatively few journals account for the most important scientific research, with 300 journals constituting 50% of all citations and 3000 journals 90% (Testa, 2009).

The suitability of using the Web of Science has been questioned within the social sciences. As discussed in the introduction, social scientists prefer to publish and cite books rather than peer-reviewed journals (e.g., Hicks, 1999; Huang & Chang, 2008; Lariviére et al., 2006; Nederhof, 2006; van Leeuwen, 2006). It has also been criticized for being dominated by English-language publications and therefore biased towards the USA and UK (Lariviére et al., 2006; van Leeuwen, 2006). However, these concerns are not relevant for the present study, where we are interested in publications and citations independent of language written by Swedish researchers, and published in peer-reviewed journals that are widely read by an international audience. Indeed, Hicks concluded that one of the Web of Science databases included in our study, the SSCI, was an effective measure when considering solely the internationally-orientated scholarly literature.^1^ Although WoS is dominated by English-language publications, WoS also includes Swedish-language publications. To confirm this, we searched WoS with the search string “LA” = Swedish, which gave 17,736 hits. Further exploring what kinds of publications these hits represent, we listed^2^ all those appearing in the last five years (2017–2021), finding that at least Sociologisk forskning, Läkartidningen, and Nordic Studies on Alcohol and Drugs are both relevant and relatively frequent outlets for the studied disciplines. One can also note that the Web of Science includes approximately 300 journals on education and 200 for each of the other five disciplines included in the study, although it must be noted that it is often hard to define a journal to a specific scientific field. Possible differences between disciplines can therefore not be attributed to the lack of inclusion of some journals, as the number of high impact journals included in the Web of Science did not differ greatly between the six disciplines included in our study. A further criticism of the Web of Science is errors and biases in its data set due to the citation habits and preferences of authors, such as misspellings and self-citations. While this may be a severe problem for the individual case, it should be sufficiently randomly spread so as to not effect citation analyses at aggregate levels (e.g., institution or discipline as in our study) (van Raan, 2005). Comparing WoS with the more inclusive Google Scholar (GS) shows that GS requires intensive manual data cleaning and quality control, but has better coverage of other types of output than journal articles (Prins et al., 2016). As we consider only journal articles, WoS is therefore better.

### Repeatability of data collection process

We controlled for the quality of the publication and citation data by having an independent reviewer redo the searches for a subset of 200 randomly selected professors and associate professors. For publication data, the results were identical for 194 researchers (97%). The difference in publication number was only one for five of these six researchers, and two for the remaining researcher. For the number of citations, the total number of citations had increased for 20 of the 200 researchers (10%) owing to the fact that the control search was conducted 1–5 months after the initial search. In the majority of cases the increase was minimal, but had in three cases increased by three to 20 citations. As the total number of publications were the same, we consider it likely that the number of citations during the period 2000–2009 had not been fully indexed in the beginning of 2010. In conclusion, it seems that these data are reliable across time and individuals, the minor discrepancies found being of no consequence for the present analyses.

### Statistical analyses

Publication and citation data are heavily skewed, because the majority of researchers have zero or few publications, and a few researchers have many. This phenomenon is found for expertise in general, and is sometimes referred to as the Matthew effect (Merton, 1988; Simonton, 2014). Log-transforming the dependent variables using log_10_(*Y* + 1) successfully improved the normality of the data and model error terms, improved homogeneity of variance, and reduced the influence of outliers (cf. Keene, 1995). This transformation has previously been found to be most appropriate for similar data (Madison & Fahlman, 2020).

We do not control for the affiliation of researchers because the present analysis is based on the whole population of researchers active in the relevant fields in Sweden, and we were interested in the Swedish research community as a whole.

For both publications and citations, we assessed the effects of discipline and academic rank through multiple regression. If the number of publications differ it is trivial that also the number of citations will differ in the same way, so we used the number of citations per publication as dependent variable. The results are generally reported as the geometric mean (*GM*—anti-log of the mean of the transformed data) and its associated 95% confidence interval (*CI*—the anti-log of the 95% CI of the transformed data). We also report the arithmetic mean (*AM*—the mean of the raw, untransformed data) for comparison, alongside geometric means in the text.

Data were collected without regard to the researchers’ previous affiliations. Therefore, publications and citations to these publications were included which may have been published under previous positions of these researchers both in Sweden and abroad. Furthermore, the data set does not include data for researchers with other titles, for example previous graduate students, guest professors and professors emeritus.

## Results

First, we consider the distribution of the data, which is described for both transformed and untransformed variables in Table 1. Noting that the minimum values are nil throughout all variables, we find that this holds across all disciplines. In other words, there are researchers in every discipline who have no publication and no citation at all, regardless of rank. This prompts a further exploration of how common this is.

**Table 1:** Descriptive summary statistics across all participants (N = 959) for total number of publications, citations, and citations per publication, with and without log transforming

	Mean	Maximum	SD	Skewness	Kurtosis
Publications	15.92	642	34.2	10.50	165.5
Log10 publications	0.8675	2.81	0.572	0.037	− 0.754
Citations	159.9	10,028	503.8	10.83	172.1
Log10 citations	1.35	4.0	1.009	0.025	− 1.242
Citations per publication	5.56	104.0	8.06	4.73	39.44
Log10 citations per publication	0.59	2.02	0.455	0.080	− 1.007

Estimates are computed across all six disciplines with a total of 959 participants, who have together authored 15,288 publications. All minimum values are 0, and are therefore excluded

**Fig. 1:**
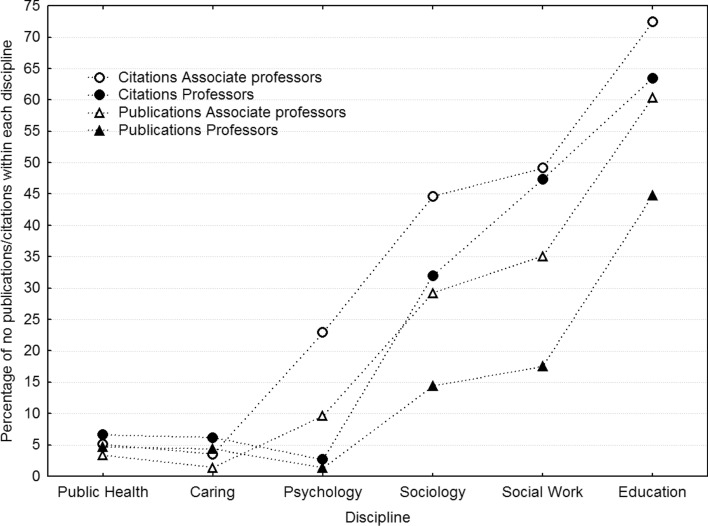
Percentage of researchers within in each discipline that have nil publications or citations in WoS, separated by academic rank, plotted in order of the mean across publications, citations and academic rank. *Caring* Nursing and Caring science

To this end, Fig. 1 plots the proportion of researchers within in each discipline that have nil publications or citations in WoS, separated by academic rank. Also, non-parametric statistics separated by discipline and rank are listed in Table 4 (“Appendix”), showing that the 25th percentile and even the median number of publications and citations are close to zero for Sociology, Social Work, and Education. Still, the maximum and 75th percentile frequencies are considerably higher, again attesting to the extreme skew and long right tails. For example the most productive professors in these three disciplines have between 20 and 78 publications and 184 to 586 citations. This remains dwarfed, however, by 132 to 506 publications and 1550 to 10 thousand citations for the most productive professors in Public Health, Nursing and Caring science, and Psychology. Nevertheless, the skewness and kurtosis estimate in Table 1 confirm our using log transformations for the following analyses.

For reference, means and SDs for both transformed and untransformed variables as a function of discipline and academic rank are listed in Table 2.

**Table 2:** Raw and transformed means and standard deviations for number of publications and number of citations as a function of discipline and rank (associate professor or professor) across participants

Discipline	Rank	N	Publ		Log10Publ		Cit		Log10Cit	
			M	SD	M	SD	M	SD	M	SD
Public health	Ass	39	17.02	15.60	1.083	0.442	160.3	224.9	1.839	0.699
	Prof	76	32.97	28.15	1.377	0.403	312.0	344.4	2.165	0.672
Caring	Ass	144	22.68	55.07	1.129	0.420	223.1	490.3	1.848	0.727
	Prof	109	34.40	54.93	1.300	0.493	455.1	1155.9	2.061	0.840
Psychology	Ass	82	10.36	10.38	0.857	0.455	91.1	151.5	1.296	0.911
	Prof	73	27.81	29.98	1.252	0.460	275.7	440.6	2.006	0.751
Sociology	Ass	64	4.86	5.80	0.558	0.444	24.9	72.78	0.713	0.760
	Prof	89	8.40	11.62	0.773	0.416	47.8	103.4	0.962	0.831
Social work	Ass	57	2.28	3.11	0.369	0.349	7.13	26.44	0.406	0.527
	Prof	49	4.30	6.30	0.524	0.398	18.12	37.75	0.626	0.738
Education	Ass	57	1.53	3.485	0.232	0.337	2.17	8.83	0.190	0.369
	Prof	120	2.27	3.73	0.338	0.362	8.68	22.89	0.406	0.610
All groups		959	15.92	33.71	0.867	0.572	159.9	503.8	1.305	1.010

*Ass* Associate professor, *Prof* Tenured professor, *Publ* number of publications, *Cit* number of citations, *Caring* Nursing and Caring science

### Number of publications

**Fig. 2:**
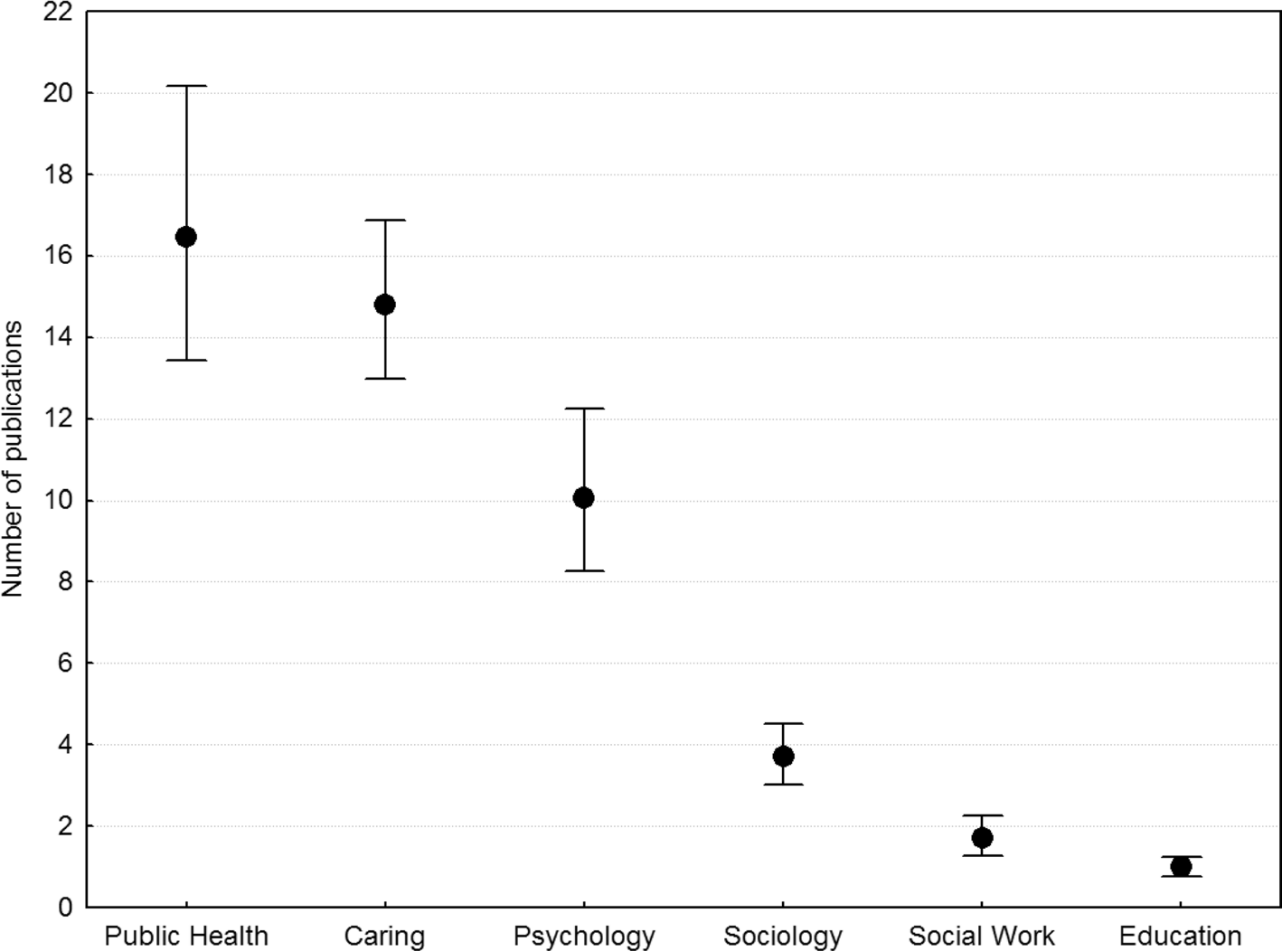
Number of publications as a function of discipline, in order of magnitude, across researchers and academic rank, plotted as geometric means and 0.95 confidence intervals. *Caring* Nursing and Caring science

Figure 2 shows the number of publications as a function of discipline. The three disciplines with the most publications were Public Health, Psychology, and Nursing and Caring science. Professors had close to 50 percent more publications (*GM* = 7.52, *CI* 6.566–8.587; *AM* = 18.97; *N* = 516) than associate professors (*GM* = 5.23, *CI* 4.55–5.98; *AM* = 12.38; *N* = 443). Multiple regression analysis (MRA) with Log_10_ publications and Log_10_ citations as dependent variables and discipline and academic rank as predictors are reported in Table 3.

**Table 3:** Results of linear regressions of discipline and academic rank (professor vs. associate professor) upon the numbers of publications and citations

	*β*	*r*^2^	*p*
Regression summary for Log_10_ publications^a^			
Intercept			< 0.000001
Discipline	0.684	0.430	< 0.000001
Academic rank	0.203	0.040	< 0.000001
Regression summary for Log_10_ citations^b^			
Intercept			< 0.000001
Discipline	0.694	0.450	< 0.000001
Academic rank	0.172	0.029	< 0.000001

^a^*N* = 959, *R* = 0.691, *R*^2^ = 0.478, Adjusted *R*^2^ = 0.476, *r*^2^ = squared semi-partial correlations

^b^*N* = 959, *R* = 0.694, *R*^2^ = 0.481, Adjusted *R*^2^ = 0.480, *r*^2^ = squared semi-partial correlations

Interactions between discipline and rank were not statistically significant when added to the models in Table 3. In terms of effect sizes, Cohen’s *d* for each successive pairwise comparisons was 0.163 for Public Health and Nursing and Caring science, 0.339 for Nursing and Caring science and Psychology, and so forth from left to right in Fig. 2: 0.776, 0.584, and 0.367. The effect size for the largest contrast, between Education and Public Health, was 2.491.

### Number of citations per publication

**Fig. 3:**
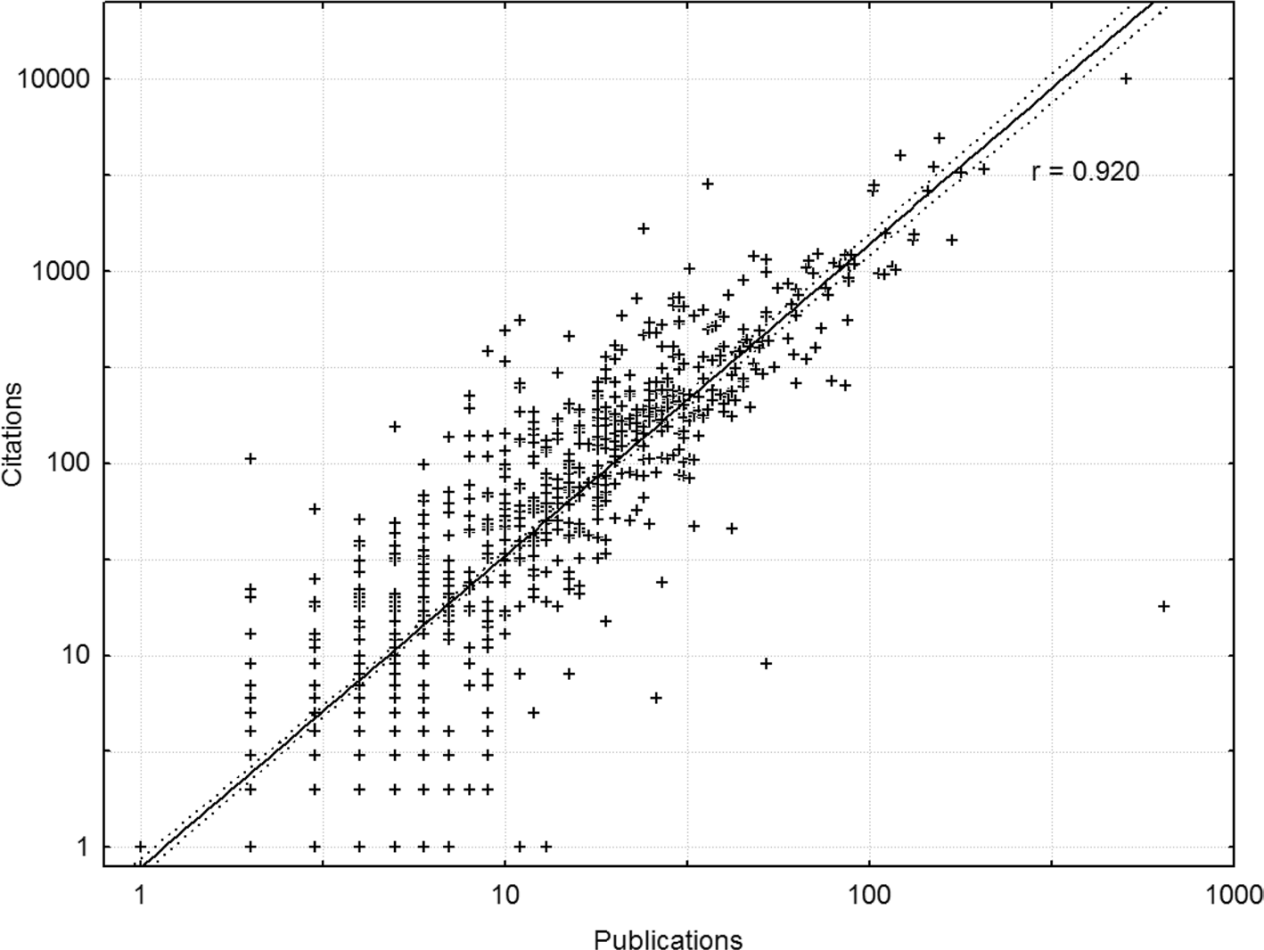
Scatterplot of the number of citations versus the number of publications. Each point is one of 959 researchers, the line represents a linear fit with 0.95 confidence bands, and the correlation is 0.92

**Fig. 4:**
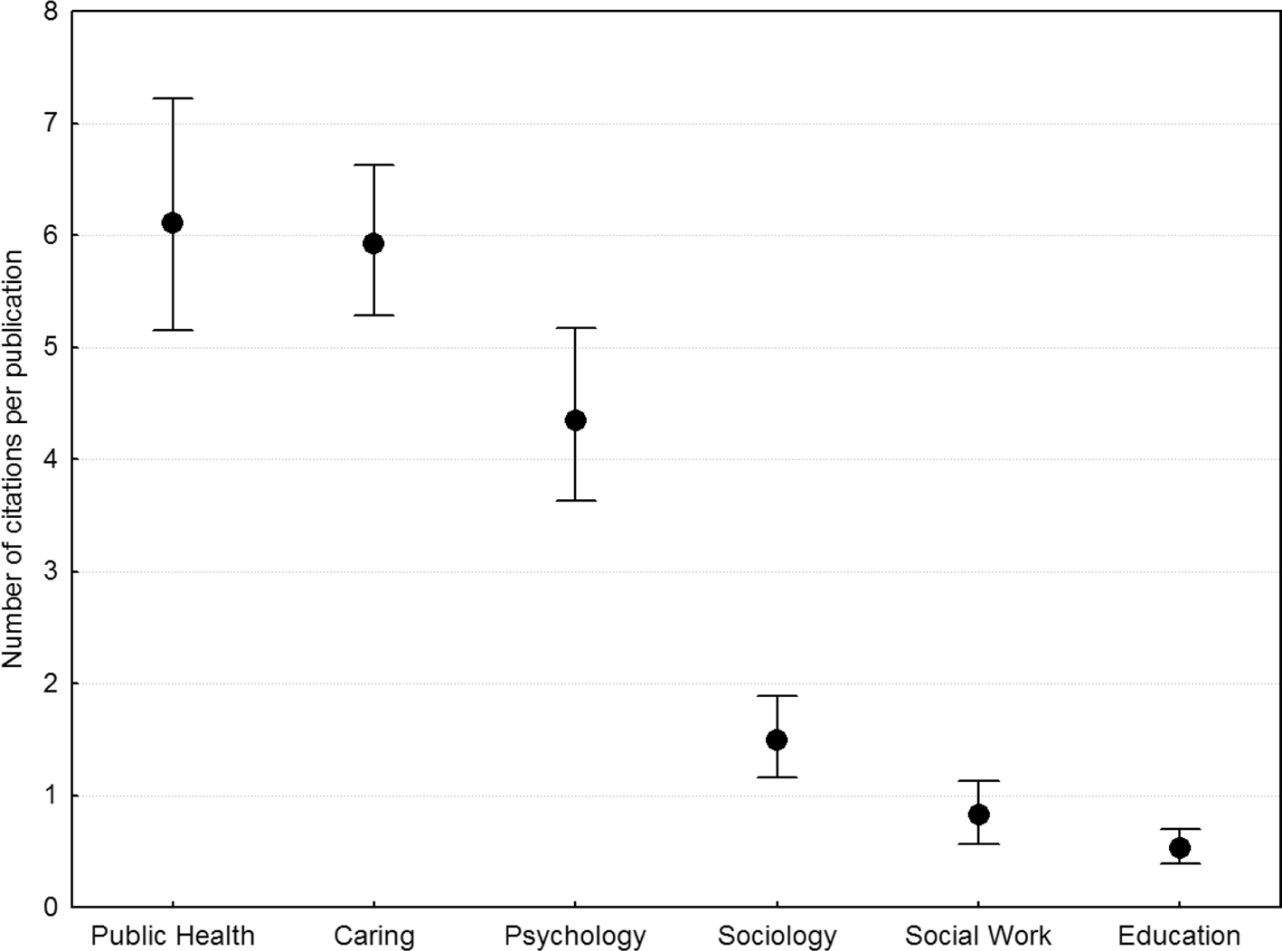
Number of citations per publication as a function of discipline, in order of magnitude, across researchers and academic rank, plotted as geometric means and 0.95 confidence intervals. *Caring* Nursing and Caring science

The log–log correlation between the numbers of publications and citations was 0.920 and is depicted in Fig. 3, showing that on average each publication generates on the order of 10 citations. Again, to control for this we used the number of citations per publication, calculated as the ratio of the total number of citations to the number of publications for each researcher. As seen in Fig. 4, the disciplines with the highest average number of citations per publication were Public Health, Psychology, and Nursing and Caring science, and professors’ publications were cited more often (*GM* = 3.07, *CI* 2.72–3.44, *AM* = 5.583, *N* = 516) than associate professors’ (*GM* = 2.67, *CI* 2.32–3.05, *AM* = 5.545, *N* = 443; *d* = 0.100). In terms of total number of citations, professors had on average 200.7 as compared to 112.4 for associate professors. Finally, there was an interaction between discipline and rank such that the magnitude of the professors’ advantage was small for Public Health and Nursing and Caring science (*d* ~ 0.05), was substantially larger and about the same for Education, Sociology, and Social Work (*d* ~ 0.3), and was largest for Psychology (*d* = 0.86).

For the number of citations per publication, Cohen’s *d* for each pairwise comparison from left to right in Fig. 4 was 0.081, 0.327, 0.800, 0.372, and 0.240, and 2.165 for the largest contrast between Education and Public Health.

**Fig. 5:**
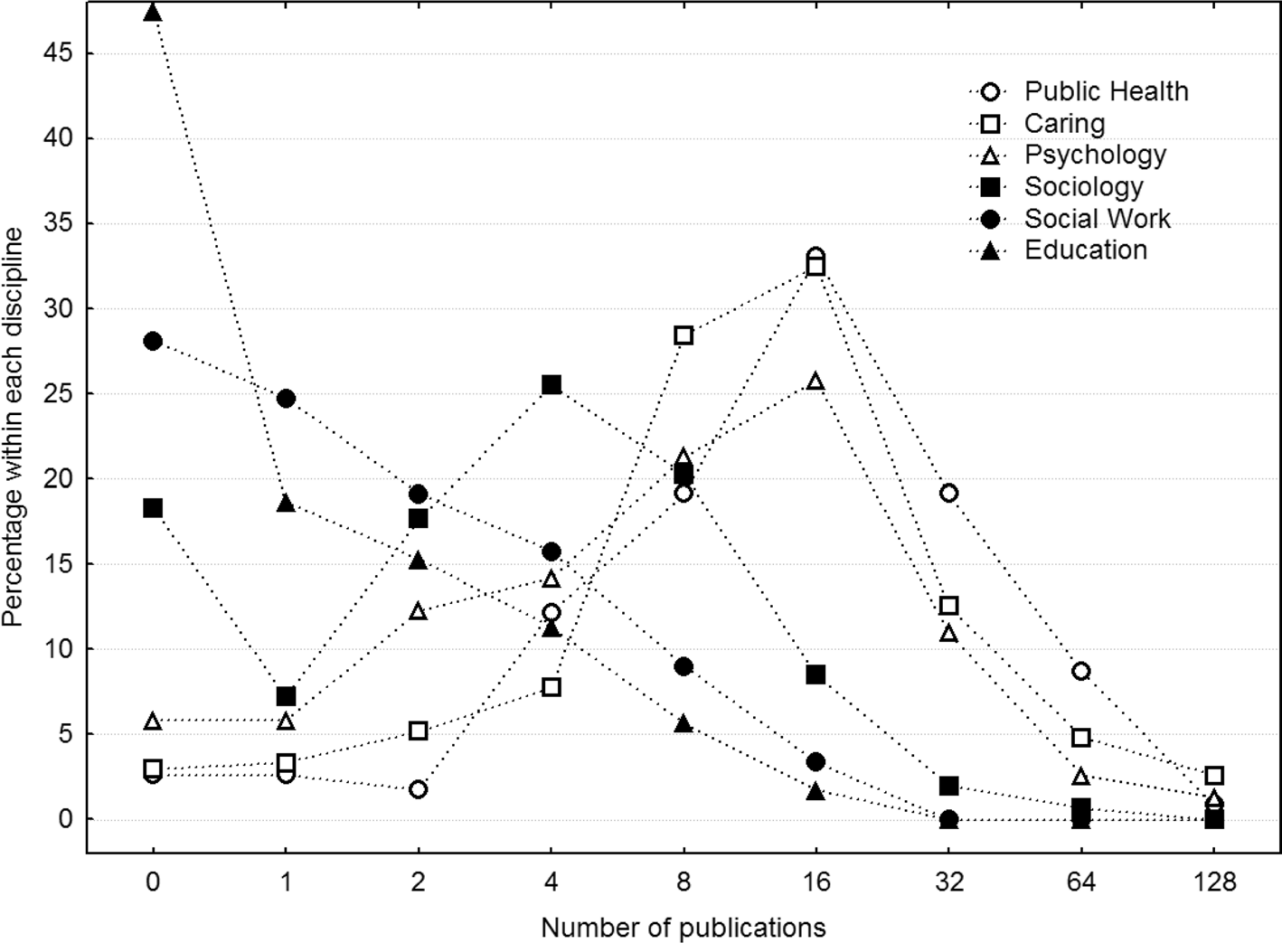
Frequency distributions of the number of publications per researcher for each discipline, binned according to a geometric scale. *Caring* Nursing and Caring science

These differences between disciplines are very large indeed, which together with the skewness and the finding that many researchers have nil publications as well as citations suggests that there might be qualitatively different researchers within each discipline. In other words, some of them might follow the general tendency to publish articles and thereby gain impact and attract citations, whereas others might publish very little or exclusively in other types of publications. To gain some insight into this, we conclude the result section by counting the number of researchers that fall into these categories. Across all disciplines, 157 researchers have nil publications (16.3%), 87 (9%) have one, and 106 (11%) have two, with no systematic differences across academic rank. Likewise, 252 researchers have nil citations (26.2%), 27 (2.8%) have one, and 41 (4.3%) have two, again with no systematic differences across academic rank. The difference between professors and associate professors begin rather to manifest at higher levels of performance, above around 15 publications and 100 citations. In numbers of individuals, 316 have more than 15 publications and 300 have more than 100 citations. To further convey these patterns of results across disciplines, the dependent variables were categorized into geometric series of powers of 2, each bin including all researchers who have numbers equal to that or smaller than the next larger category. For example, the category 4 includes 4–7, 16 includes 16–31, and 512 includes 512–1023. These frequencies are then divided by the total number of researchers in that discipline, thus expressing the relative frequency distribution of researchers within each discipline as a function of the dependent variables along an exponential scale. The result for the number of publications in Fig. 5 does not exhibit any bimodal distribution, and hence no strong support for qualitative differences across researchers. Rather, they all express a modal tendency, although this mode is quite different: Nil for Education and Social Work, four for Sociology, and 16 for the remaining disciplines. If anything, these three higher-performing disciplines express a tendency for qualitative differences, in that about 2–12% have fewer than four publications. This corresponds to 57 out of 541 individuals, or just over 10%. Twenty-one of these belong to the professor category, and it is notable that they have apparently merited themselves for professorships although these disciplines rely heavily on peer-reviewed publications as merits.

**Fig. 6:**
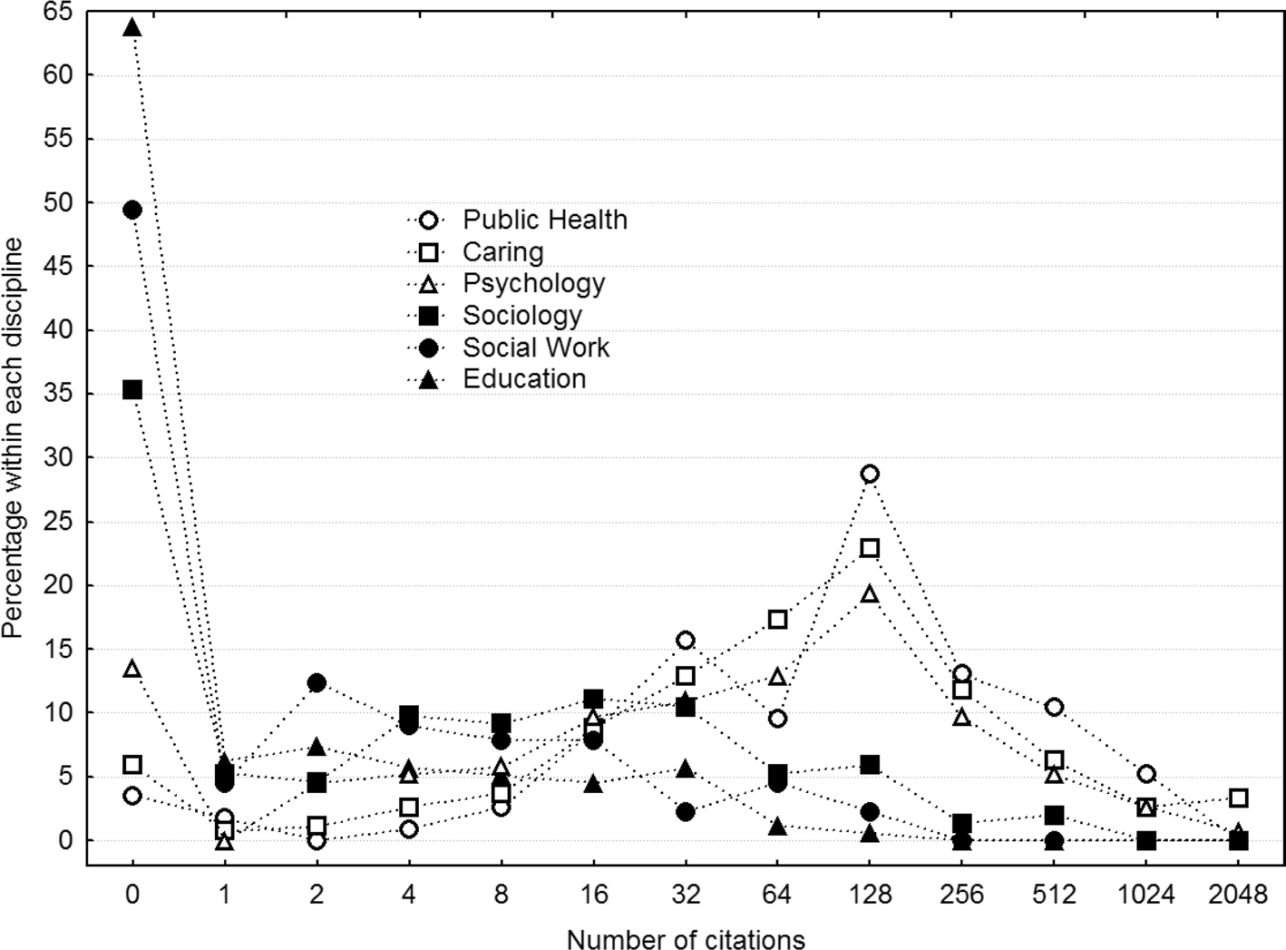
Frequency distributions of the number of citations per researcher for each discipline, binned according to a geometric scale. *Caring* Nursing and Caring science

The citations in Fig. 6 do support a qualitative difference, however, in that the by far largest relative proportion of researchers in Education, Sociology, and Social Work are not cited even once, ranging from 35 to 64%. Apart from that, they do not exhibit any clear mode, again with the exception of Sociology, which suggests a mode at 16 citations. In contrast, Public Health, Psychology, and Nursing and Caring science all have modes at around 128 with substantially higher frequencies.

## Discussion

In the present study, we have described differences in the relative amount of research dissemination through peer-reviewed journal articles across disciplines that ostensibly provide similar knowledge and hence serve the same communities. We document differences close to one order of magnitude between the highest- and lowest performing disciplines, with medium-to-large effect sizes between 0.25 and 2.6, except for Public Health and Nursing and Caring science, who were quite similar.

While professors do publish more than associate professors, this is only the case for the more productive one-third of researchers. This is consistent with the relatively low level of public contractual funding in Sweden (Öquist & Benner, 2015), given a substantial association between research productivity and factors related to being a principal investigator, in terms of receiving external funding and running a laboratory. Factors that are positively associated with research dissemination include the intensity and quality of colleagues’ research and the number of foreign post-doctoral researchers in the lab, while the size of the lab is negatively associated (Carayol & Matt, 2006). However, we note that our figures are aggregated across time, and that more accurate effects of academic rank would need to control for the number of years that each researcher has been conducting qualified research.

The main strength of the present study is its representativeness, in that it obtains the full population of researchers active in these fields of research, and is in other words based on individuals rather than publications or journals. We also provide detailed descriptive statistics. One weakness is that we do not consider secular trends, the reason being that such trends are strongly confounded by the researchers themselves, as they tend to be active across several decades and to maintain their publication preferences across their academic life. It would require huge data sets spanning such long periods that many confounding variables would play a significant role, as for example the constantly increasing rate of publishing and the emergence of fashionable sub-disciplines within main disciplines (Söderlund & Madison, 2015). Another concern may be that the actual metrics are dated ten years ago, but we maintain that the comparison is nevertheless relevant. First, using population-based data and this detailed level of analysis provides a unique snapshot of the relative amount of journal dissemination of the six disciplines. Second, traditions die hard, and there are no indications of significant relative changes amongst these disciplines until the present. This also raises the issue that trends across countries and across time are poorly captured by the literature because, like the present study, it typically consists of snapshots of a given set of disciplines. Future research would have to apply longitudinal designs to address this, as well as international comparative data (e.g., Dutton et al., 2020; van der Linden et al., 2020).

There has been a trend in the social sciences for increased publishing in peer-reviewed journals across several decades (Lariviére et al., 2006). Yet, our data demonstrate large differences amongst the studied disciplines, suggesting that adoption of an international publication culture is not evident in our data. This is especially pronounced in education, social work and sociology, a pattern that can be seen worldwide (e.g., Huang & Chang, 2008; Ingwersen, 2000; Lariviére et al., 2006; Lindholm-Romantschuk & Warner, 1996; Schaffer, 2004). Our study is based on 10-year-old data. As there are no other similar but younger studies to our knowledge, we cannot be sure if a change has occurred amongst the disciplines. However, an inventory (Sundell & Olsson, 2021) of all Swedish outcome research of behavioral, psychological and social interventions during 1990–2019 indicate that no significant change has occurred when it comes to this type of research within the disciplines of sociology and social work; During 2010 to 2019 only 4 out of 437 trials was published by a researcher from sociology or social work, while psychology, public health and nursing and caring science rapidly increased their number of trials from 19 (1990–1999), to 61 (2000–2009) and 228 (2010–2019). If this result is representative or not, future investigations will reveal.

Despite the well-known disadvantages of peer-review (Lee et al., 2013; Thurner & Hanel, 2011; Wicherts, 2017), publication in international peer-reviewed journals is widely accepted as the best system currently in place for the dissemination, critical review, discussion, and thereby development of knowledge (e.g., Balietti et al., 2015; Lawrence, 2008; Righi & Takacs, 2017). We believe that a shift towards peer-reviewed articles also in education, social work, and sociology would equally benefit both applied and conceptual studies. Even novel theories have to take into account what has been proposed earlier and the breadth and limitations of the theory’s implications. Furthermore, it will ensure that new theories and results are exposed to critical review, something which is especially important in theoretically homogenous disciplines, which has been suggested to be the case for education and social work in Sweden (Eklund, 2000). In summary, the quality of any form of knowledge produced can only improve with exposure to international peer-review process.

This said, we do not imply that other sources of knowledge dissemination (e.g., books or newspaper articles) are of less value to society or should occur less frequently. Each type of publication serves somewhat different purposes. We refer here to the development, dissemination and quality of knowledge. International publication will not only improve the quality of knowledge produced, but also the accessibility of results for more geographically isolated groups interested in similar social issues. Future work should monitor the trends across disciplines, but there may also be a call for changing policies within academe.

## Appendix

**Table 4:** Medians and quartiles for number of publications and number of citations as a function of discipline and rank (associate professor or professor)

Discipline	Rank	Number of publications					Number of citations			
		N	Md	Max	Q25	Q75	Md	Max	Q25	Q75
Public health	Ass	39	14	85	6	26	117	1215	26	221
	Prof	76	25	132	14	42	189.5	1550	59	413
Caring	Ass	144	12	642	8	21	86	3502	30	197
	Prof	109	22	506	12	36	163.5	10,028	47	360
Psychology	Ass	82	7	59	2	16	26	862	2	132
	Prof	73	18	168	9	38	142	2817	41	289
Sociology	Ass	64	3	26	0	7.5	2	555	0	24.5
	Prof	89	5	78	2	9	7	586	0	42
Social work	Ass	57	1	15	0	3	0	184	0	4
	Prof	49	2	29	1	5.5	1.5	184	0	16
Education	Ass	57	0	23	0	2	0	65	0	1
	Prof	120	1	20	0	3	0	198	0	5
All groups		959	7	642	1	19	25.5	10,028	0	146

*Ass* Associate professor, *Prof* Tenured professor, *Caring* Nursing and caring science, All minimum values are 0, and are therefore not shown in the table

## Footnotes

1 At the time the searches were conducted, the Web of Science included the following databases: Science Citation Index Expanded (SCI-Expanded, 1899–present), Social Sciences Citation Index (SSCI, 1899–present), Arts and Humanities Citation Index (A&HCI, 1975–present), Conference Proceedings Citation Index-Science (CPCI-S, 1990–present), and Conference Proceedings Citation Index-Social Science and Humanities (CPCI-SSH, 1990–present).

2 Ekonomiska Samfundets Tidskrift, Ethnologia Scandiavica, European Journal of Scandinavian Studies, Fornvännen, Historisk tidskrift, Internasjonal politikk, Konsthistorisk tidskrift, Laeknabladid, Läkartidningen, Moderna språk, Nordic Journal of Digital Literacy, Nordic Studies on Alcohol and Drugs, Scandia, Scandinavian Jewish Studies, Skandiavskaya Filologiya Sociologisk forskning, Tidskrift för den Norske Laegeförening, Tidskrift för samfunnsforskning, World Literature Today.

## References

[CR1] Balietti S, Mäs M, Helbing D (2015). On disciplinary fragmentation and scientific progress. PLoS ONE.

[CR2] Bonaccorsi A, Daraio C, Fantoni S, Folli V, Leonetti M, Ruocco G (2017). Do social sciences and humanities behave like life and hard sciences?. Scientometrics.

[CR3] Bornmann L, Thor A, Marx W, Schier H (2016). The application of bibliometrics to research evaluation in the humanities and social sciences: An exploratory study using normalized Google Scholar data for the publications of a research institute. Journal of the Association for Information Science and Technology.

[CR4] Carayol N, Matt M (2006). Individual and collective determinants of academic scientists' productivity. Information Economics and Policy.

[CR5] Chi P-S (2016). Differing disciplinary citation concentration patterns of book and journal literature?. Journal of Informetrics.

[CR6] de Rijcke S, Wouters PF, Rushforth AD, Franssen TP, Hammarfelt B (2016). Evaluation practices and effects of indicator use: A literature review. Research Evaluation.

[CR7] Dutton E, van der Linden D, Madison G (2020). Why do high IQ societies differ in intellectual acheivement? The role of schizophrenia and left-handedness in per capita scientific publications and Nobel prizes. Journal of Creative Behavior.

[CR8] Eklund H (2000). Vart är pedagogikforskningen på väg? Ämnesområden och forskningsmönster i svenska doktorsavhandlingar under en femårs-period (Where is education research headed? Topic areas and research patterns in Swedish doctoral theses under a five-year period. Pedagogisk Forskning i Sverige.

[CR9] Fanelli D, Lariviére V (2016). Researchers' individual publication rate has not increased in a century. PLoS ONE.

[CR10] Hicks D (1999). The difficulty of achieving full coverage of international social science literature and the bibliometric consequences. Scientometrics.

[CR11] Honoré P, Wright D, Berwick DM, Clancy CM, Lee P, Nowinski J (2011). Creating a framework for getting quality into the public health system. Health Affairs.

[CR12] Huang M, Chang Y (2008). Characteristics of research output in social sciences and humanities: From a research evaluation perspective. Journal of the American Society for Information Science and Technology.

[CR13] Ingwersen P (2000). The international visibility and citation impact of Scandinavian research articles in selected social science fields: The decay of a myth. Scientometrics.

[CR14] Jokic M, Mervar A, Mateljan S (2019). Comparative analysis of book citations in social science journals by Central and Eastern European authors. Scientometrics.

[CR15] Keene ON (1995). The log-transform is special. Statistics in Medicine.

[CR16] Kousha K, Thelwall M (2009). Google book search: Citation analysis for social science and the humanities. Journal of the Association for Information Science and Technology.

[CR17] Kulczycki E, Engels TCE, Pölönen J, Bruun K, Duskova M, Guns R (2018). Publication patterns in the social sciences and humanities: Evidence from eight European countries. Scientometrics.

[CR18] Kyvik S (2003). Changing trends in publishing behaviour among university faculty, 1980–2000. Scientometrics.

[CR19] Lariviére V, Archambault E, Gingras Y, Vignola-Gagne E (2006). The place of serials in referencing practices: Comparing natural sciences and engineering with social sciences and humanities. Journal of the American Society for Information Science and Technology.

[CR20] Lawrence PA (2008). Lost in publication: How measurement harms science. Ethics in Science and Environmental Politics.

[CR21] Lee CJ, Sugimoto CR, Zhang G, Cronin B (2013). Bias in peer review. Journal of the American Society for Information Science and Technology.

[CR22] Lindholm-Romantschuk Y, Warner J (1996). The role of monographs in scholarly communication: An empirical study of philosophy, sociology and economics. Journal of Documentation.

[CR23] Madison G, Fahlman P (2020). Sex differences in the number of scientific publications and citations when attaining the rank of professor in Sweden. Studies in Higher Education..

[CR24] Merton RK (1988). The Matthew Effect in science: II. Cumulative advantage and the symbolism of intellectual property. Isis.

[CR25] National Forum on Health (1997). Canada health action: Building on the legacy.

[CR26] Nederhof AJ (2006). Bibliometric monitoring of research performance in the social sciences and the humanities: A review. Scientometrics.

[CR27] Olsson TM, Sundell K (2015). Research that guides practice: Outcome research in Swedish PhD theses across seven disciplines 1997–2012. Prevention Science.

[CR28] Öquist G, Benner M, Welpe IM, Wollersheim J, Ringelhan S, Osterloh M (2015). Why are some nations more successful than others in research impact? A comparison between Denmark and Sweden. Incentives and Performance. Governance of Research Organizations.

[CR29] Prins AAM, Costas R, van Leeuwen TN, Wouters PF (2016). Using google scholar in research evaluation of humanities and social science programs: A comparison with web of science data. Research Evaluation.

[CR30] Puuska H-M (2010). Effects of scholar's gender and professional position on publishing productivity in different publication types Analysis of a Finnish University. Scientometrics.

[CR31] Righi S, Takacs K (2017). The miracle of peer review and development in science: An agent-based model. Scientometrics.

[CR32] Schaffer T (2004). Psychology citations revisited: Behavioral research in the age of electronic resources. Journal of Academic Librarianship.

[CR33] Simonton DK (2014). Creative performance, expertise acquisition, individual differences, and developmental antecedents: An integrative research agenda. Intelligence.

[CR34] Söderlund T, Madison G (2015). Characteristics of gender studies publications: A bibliometric analysis based on a Swedish population database. Scientometrics.

[CR35] Sundell, K. (2010). *Internationella publikationer och citeringar under perioden 2000–2009 hos svenska professorer och docenter inom folkhälsovetenskap, omvårdnadsvetenskap, pedagogik, psykologi, socialt arbete och sociologi*. Socialstyrelsen.

[CR36] Sundell K, Åhsberg E (2016). Trends in methodological quality in controlled trials of psychological and social interventions. Research on Social Work Practice.

[CR37] Sundell K, Olsson TM, Mullen EJ (2017). Social intervention research. Oxford bibliographies in social work.

[CR38] Sundell K, Olsson TM (2021). Svenska Effektutvärderingar av Beteendemässiga, Psykologiska och Sociala Insatser 1990–2019.

[CR39] Testa J (2009). The Thomson Reuters journal selection process. Transnational Corporations Review.

[CR40] Thurner S, Hanel R (2011). Peer-review in a world with rational scientists: Toward selection of the average. European Physical Journal B.

[CR41] van der Linden D, Dutton E, Madison G (2020). National-level indicators of androgens are related to the global distribution of scientific productivity and Nobel prizes. Journal of Creative Behavior.

[CR42] van Leeuwen TN (2006). Using google scholar in research evaluation of humanities and social science programs: A comparison with web of science data. Scientometrics.

[CR43] van Leeuwen TN (2013). Bibliometric research evaluations, Web of Science and the Social Sciences and Humanities: A problematic relationship?. Bibliometrie Praxis Und Forschung.

[CR44] van Raan AFJ (2005). Fatal attraction: Conceptual and methodological problems in the ranking of universities by bibliometric methods. Scientometrics.

[CR45] Wicherts JM (2017). The weak spots in contemporary science (and how to fix them). Animals.

